# Chemotherapy casted shadow: Mees’ and Beau's lines

**DOI:** 10.1002/jha2.468

**Published:** 2022-05-09

**Authors:** Abdulaziz Altwijri, Nourah Faden, Mansour Alfayez

**Affiliations:** ^1^ Department of Hematology King Fahad Medical City Riyadh Saudi Arabia

**Keywords:** Beau's lines, chemotherapy toxicity, DLBCL, Mees’ lines, RCHOP

1

A 28‐year‐old female received six cycles of rituximab, cyclophosphamide, doxorubicin, vincristine, and prednisone chemotherapy to treat diffuse large B‐cell lymphoma. She completed her treatment (see Figure 1) 3 weeks before this photograph. Examination showed multiple white lines on the fingernails, known as Mees’ lines. Mees’ lines are transverse, nonblanching white bands that run parallel to the lunula around the entire nail bed. The lines matched the start of each cycle, with the distance between the lines representing the time interval between cycles. In this patient, four lines can be identified with consistent intervals on the body of her nails, while the fifth and sixth lines are seen at a more significant distance at the nail‐free edge. This might be related to treatment delay after cycle 2 (40 days) due to intensive care unit admission with sepsis and coronavirus disease (COVID‐19) pneumonia. In the third and fourth cycles (the outer lines on the nail bed), we can see roughness and indentation (on the left‐hand thumb and index finger), representing Beau's lines. Beau's lines are transverse depressions in the nail plate caused by transient cell division interruption in the proximal nail matrix. Both lines are commonly seen in patients with a history of chemotherapy exposure.

**FIGURE 1:**
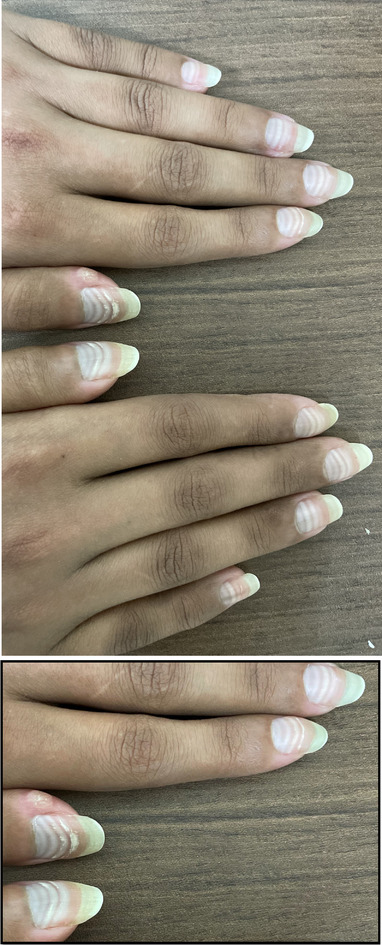
Multiple white lines on the fingernails known as Mees’ lines, and a few transverse depressions (on the left‐hand thumb and index finger) represent Beau's lines

## CONFLICT OF INTEREST

The authors declare they have no conflicts of interest.

## ETHICS STATEMENT

The photograph was taken with the patient's informed consent and in accordance with the declaration of Helsinki

## FUNDING INFORMATION

The authors received no specific funding for this work.

## PATIENT CONSENT STATEMENT

Informed consent was obtained.

## Data Availability

The authors declare that the data supporting the findings of this report are available within the article.

